# The Mediating Role of Rape Myths in the Relationship Between the Use of Hentai Pornography and Sexually Aggressive Strategies: A Study with College Students

**DOI:** 10.1177/08862605241286004

**Published:** 2024-10-10

**Authors:** Beatriz Almeida, Hugo Gomes, Joana Carvalho

**Affiliations:** 1 Faculty of Psychology and Educational Sciences, University of Porto, Portugal; 2 Human Development and Violence Research Centre, Federal University of Pelotas, Brazil; 3 William James Center for Research, Department of Education and Psychology, University of Aveiro, Portugal

**Keywords:** sexual aggression, rape myths, hentai, pornography, college students

## Abstract

The current study aims to test the hypothetical link between hentai pornography use, rape myths endorsement, and sexually aggressive strategies. Furthermore, it aims to capture if such a trajectory echoes across gender and human-realistic pornography. The work targeted a sample of 906 college students, of whom 533 were men and 373 were women, who completed an online survey to capture pornography use, rape myths, and sexually aggressive behavior. Participation criteria included being heterosexual, over 18, pursuing higher education, and using hentai pornography. The results revealed that the frequency of hentai pornography use predicts sexually aggressive behaviors in male and female participants. The endorsement of rape myths mediated the relationship between the frequency of hentai use and sexually aggressive behavior in both genders and the relationship between the intensity of hentai use and sexually aggressive behavior in men. When considering human-realistic pornography, it was found that the frequency of use predicts sexually aggressive behavior in female participants. The endorsement of rape myths mediated the relationship; no other relationship was found. This work aims to increase awareness about the implications of using hentai and human-realistic pornography and highlight the importance of sexual violence prevention in the college setting.

## Introduction

The growth in technology and Internet services is connected to the fast distribution of pornography and its associated problems, according to studies in the field (Foubert et al., 2011; Kazue, 2016; Kunaharan et al., 2020; Walker et al., 2016). The anonymity and accessibility of the Internet seemed to be the perfect grounds for pornographic content to flourish and it became a market with high profits for whoever engaged in it (Dines, 2011). This easy commodity has allowed for new content that pushed the limits of what is legal and morally acceptable, with little repercussions (Kunaharan et al., 2020).

Pornography has centered debates around its morality and benefits for society with the general population. In investigation, researchers wonder about its effects on consumers. Foubert et al. (2011) argues that this new and edgier content seeks to satisfy the evolution of society’s sexual needs, being described as more extreme than it was as far as two decades ago (Foubert et al., 2011).

However, just as there are arguments favoring the negative effects of pornography, there are also views that highlight the neutral and even positive aspects of pornography. It may simply be a visual record of sex that people can choose to watch. It may even be a safe place to explore their sexuality beyond the heteronormative and more conservative conceptions of sex commonly shown in mainstream media (Harrison & Ogden, 2018).

The World Health Association classifies sexual aggression as “any sexual act, attempts to obtain [it], or other act directed against a person’s sexuality using coercion, by any person regardless of their relationship to the victim, in any setting (. . .)” (World Health Organization, 2021).

Sexual aggression is believed to be particularly high among college students (Moreira et al., 2022), as it is a more prevalent crime at university when compared to other crimes (RAINN, n.d.). In a descriptive study, Abbey et al. (2001) reported that one-third of American male college students had perpetrated some form of sexual assault in the dating context. A survey conducted by the Association of American Universities (2019) reports that 13% of the nearly 182,000 students surveyed had experienced non-consensual sexual interactions, with 25.9% of women surveyed reporting such interactions. In Portugal, a survey by the Federação Académica de Lisboa (2019) of 932 valid cases of college students living in the capital city concluded that 34.2% (318) of these students reported having been victims of sexual violence with physical contact at least once. It is important to note, as well, that 23.29% of perpetrators were also university students, and 18.92% were staff from the universities the victims attended (Federação Académica de Lisboa, 2019). Statistics like these have led to the widespread notion that sexual violence on college campuses is an epidemic (Brown, 2020; Dolce, 2020; Tchen & Jarrett, 2017).

Since the beginning of the scientific study of human sexuality, pornography consumption has been seen as a risk factor for sexual aggression, specifically sexual aggression against women (Ferguson & Hartley, 2009). While there is considerable research on the relationship between pornography and sexually aggressive outlets, the outcomes are mixed.

Those who find a correlation between pornography consumption and sexual offending outlets usually report small statistically significant effects (Ferguson & Hartley, 2022). Nevertheless, Wright et al. (2016) and Hald et al. (2010) found, in their meta-analyses, that pornography appears associated with an increased likelihood of enacting sexual aggression, as well as more violence-supportive attitudes. Similarly, Burgess (2007) and Foubert et al. (2011) observed the same phenomenon in college-aged men. More specifically, Hald et al. (2010) and Foubert et al. (2011) claim that it is the hardcore type of pornography, which features aggressive themes, that has a stronger association with attitudes supporting violence against women.

The increase in exposure to progressively shocking pornographic content is expected to affect people’s emotions and actions (Kunaharan et al., 2020). According to Carvalho and Rosa (2020), men displayed more favorable emotions than women when watching footage featuring non-consensual sexual acts against women. Furthermore, the participants’ pupil dilation observations indicated that they experienced partial habituation after viewing that specific material. Former research (Linz, 1985) proposes that overexposure to pornography, both violent and otherwise, may lead to desensitization, or habituation, which can subsequently raise the likelihood of accepting sexual violence.

Malamuth et al. (2021) have tested their Confluence model, which consists of 4 interrelated pillars of sexual violence risk factors, in a sample of college students. The results supported all key pillars, including the fourth pillar, which designates extreme pornography use as a risk factor for sexual violence. The term “extreme” referred to its themes, centered around rape and murder, and also the frequency in which the user consumed pornography. Taken together, these studies support the notion that the relationship between the use of pornography and sexually aggressive attitudes is a complicated one that needs to be studied further, particularly regarding the themes present in pornography. This work will address this question, focusing on a specific type of pornography, namely hentai.

Due to the freedom of expression and anonymity inherent in the use of the Internet, unusual sexual interests have become increasingly normalized and more common in pornography (Nixon & Düsterhöft, 2017). This may be the case for animated pornography, or hentai, and the themes present (Nixon & Düsterhöft, 2017).

To date, hentai has received scant attention in the research literature. However, according to the yearly categorical analysis done by one of the most popular pornography distribution websites, Pornhub, hentai was the most searched term in 2021 and 2022, and a top ten search in almost every country (Pornhub Insights, 2021, 2022), showing its undeniable popularity worldwide. The same document revealed that pornography users aged 18 to 24 (Generation Z) view 72% more pornography containing cosplay (dressing up as fictional characters), 58% more cartoon pornography, and 54% more uncensored hentai pornography than other ages/generations (Pornhub Insights, 2022). This fact alludes to the target audience of hentai pornography comprising people of college age, which is the population chosen for this work.

There are different definitions of hentai, depending on the context in which this Japanese word is used, and who is using it. In Japanese, it translates to “transformation, abnormality, metamorphosis, and perversion,” but it is also an abbreviation for “hentai *seiyoku*” which translates to “abnormal sexual desires” (McLelland, 2006). Consequently, Japanese people use this word when referring to the most perverted and extreme animated content of a sexual nature, having other words to refer to basic animated pornography—“H” or *etchi*, *ero*, 18-kin, and *seijin* or *adoruto* (Ortega-Brena, 2009).

However, with the migration of Japanese animation to the West, non-Japanese people have generalized the term to mean any Japanese animation, whether visual (anime) or written (manga), with a sexual nature. As a result, the term has become a loanword rather than a translation (McLelland, 2006). However, other authors define hentai as any type of sexual animation, especially one that features perverse bodies and situations, regardless of its geographical origins (Paasonen, 2017). In this work, the latter definition will be used, that is, non-Japanese computer-generated cartoon pornography will be considered hentai as well, to facilitate the completion of the questionnaire.

Fundamentally, hentai is a genre known for its fantastic or occult narratives, which offer endless possibilities due to the lack of human limitations (Ortega-Brena, 2009; Paasonen, 2017). Unlike human pornography, hentai features fantastical or monstrous creatures, impossible bodies, and unrealistic situations often inspired by science fiction (Josephy-Hernández, 2015). Moreover, Harrington and Neilson (2009) argue that hentai also has a predilection for the pain inflicted on the female body. Ortega-Brena (2009) suggests that the preference for animated pornography can stem from the thrill of being able to suspend disbelief and enjoy impossible, borderline illegal scenarios that live pornography cannot provide access to easily.

In Japan, the birthplace of the hentai genre and the most prevalent country in producing animated pornography (Henderson, 2005), there is a growing concern from legal fronts with the prevalence and easy accessibility of pornographic videogames and cartoons featuring rape, stalking, and the molestation of women and girls, which they believe normalizes sexual violence (Kazue, 2016).

As previously stated, hentai has successfully left Japan and been marketed worldwide. As a result, some countries have decided to censor the darker parts of the pornographic genre or ban some of it altogether. However, in today’s Internet-dominated world, it is easy to bypass legal barriers and access this content as fans become unofficial distributors and upload it, sometimes even with subtitles and without censorship (Josephy-Hernández, 2015).

Given the above, it is reasonable to assume that increased use of hentai pornography could facilitate sexually aggressive behavior. Certain stable risk factors have been found to support the likelihood of sexually aggressive behavior, such as interpersonal functioning, distorted attitudes toward the crime or the victim, permissiveness toward sexual aggression, psychopathology, and maladaptive personality (Carvalho & Nobre, 2013). These are often found not only in forensic samples but also in samples of college students (Carvalho & Nobre, 2013). In this perspective, it is thought that the relationship between the use of sexually aggressive strategies and the use of hentai pornography may be mediated by the belief in rape-tolerant views, also known as rape myths.

Burt (1980) defined rape myths as “prejudicial, stereotyped, or false beliefs about rape, rape victims, and rapists” (p. 217). Offenders may benefit from adhering to certain rape myths to rationalize and maintain their aggressive actions (Wright et al., 2016). These myths tend to shift the blame from the aggressor to the victim (Burgess, 2007), as well as distort what qualifies as rape and who can be considered a credible victim (Walfield, 2021).

Assessing the acceptance of rape myths in college students seems to be key in understanding the perpetration of sexual aggression by this population, as adhering to these beliefs can be functional for sexually aggressive individuals (Burgess, 2007). Understanding one’s attitudes toward sexual violence may help comprehend their behavior toward victims and aggressors (Martins et al., 2012).

Foubert et al. (2011) found a positive correlation between the use of pornography and the acceptance of rape myths, especially the most hardcore and degrading genres of pornography, like sadomasochism and rape. Belief in rape myths is also associated with attitudes supporting violence against women (Hald et al., 2010; Malamuth et al., 2012), which have already been found in pornography users (Burgess, 2007; Foubert et al., 2011), and with sexual aggression in a study by Forbes et al. (2004). Common beliefs are blaming the victim, minimizing the psychological impact of the crime, and justifying aggressive behavior (Martins et al., 2012).

Overall, current literature suggests that there may be associations between the three key variables: the use of hentai pornography, sexually aggressive behaviors, and acceptance of rape myths. Accordingly, the current study aimed to analyze the discrepancies among the academic sources discussed in the literature review regarding the relationship between these variables. This research intends to expand on hentai pornography as a phenomenon that has not been thoroughly studied when compared to human/realistic pornography. Additionally, it seeks to examine a population with high incidences of sexual violence, which belongs within the age group of the target audience of hentai pornography.

This work raised the question of the possible existence of a mediation relationship between the three variables and investigated whether using hentai pornography predicted engagement in sexually aggressive strategies among male and female college students who consumed this type of pornography and if the relationship varied depending on how frequently and how intensely they used it. It also examined whether the endorsement of rape myths mediates such relationships. The following hypotheses were tested: The frequency and intensity of hentai pornography use predict sexually aggressive tendencies in male and female participants who have used hentai pornography (H1); this relationship is mediated by the endorsement of rape myths in men and women (H2). Even though this study is framed and focused on the hentai literature, we considered the trajectories of human/realistic pornography use, rape myths, and sexually aggressive behavior to provide a completer and more contrasting picture.

## Methods

### Participants

A non-probability sampling method was used - voluntary response sampling—to obtain a sample of 3,663 participants. Due to the high level of incomplete survey entries, it was chosen that participants who did not complete our survey protocol would be considered dropouts and thus removed. The sample then consisted of 1,583 participants.

The study’s inclusion criteria required participants to be identified as heterosexual, since the Sexually Aggressive Behaviors Scale (SABS) has only been validated for that population, currently pursuing a higher education, over 18 years old, and have used hentai pornography. As such, 50 cases were excluded from the data analysis due to not fulfilling these criteria. Furthermore, three cases were removed due to low-quality answers, for example, Internet trolls.

According to Bennett (2001), cases where we have many missing values (i.e., more than 10% missing values) negatively influence results. As such, we removed those with more than 10% missing values, equivalent to 18 participants.

Considering only the subjects who reported having watched hentai in the past, or presently, since that is the group that will be studied, the final sample consisted of 906 participants (58.8% of the total sample).

The participants included 533 identifying with the male gender and 373 with the female gender. The mean age was 22.15 (*SD* = 5.12). More than half, 65.2% (*n* = 588), were enrolled in a bachelor’s degree. See Table 1 for in-depth sociodemographic statistics.

**Table 1.** Participant’s Sociodemographic Characteristics (*n* = 906).

Variable	*n*	%
Gender		
Female	533	58.8
Male	373	41.2
Age (*M*/*SD*)	22.15	5.12
Education		
Bachelor’s degree	588	65.2
Master’s degree	253	28.1
Doctorate degree	31	3.4
Other	30	3.3

The respondents completed a web survey advertised as “sexuality in college students.” The study was distributed online, through Portuguese institutional mailing lists, social media, and survey-sharing websites.

Participants were required to provide informed consent prior to proceeding with the questionnaire. Participation was voluntary, and no personal or identifiable information was collected, ensuring confidentiality. The survey took about 15 min to complete and was available online using a survey tool, Qualtrics, Washington, USA.

After completing the survey, participants were thanked for their collaboration but were not debriefed. They received no compensation for their participation in the study.

The research protocol of this study was approved by the Ethics Committee of the Faculty of Psychology and Educational Sciences of the University of Porto in 2022.

### Measures

#### Pornography

Previously to the pool of questions, participants were given a brief explanation of what constitutes hentai and human/realistic pornography, with a set of questions dedicated to registering each use.

Human pornography was defined as “intentionally viewing, reading or listening to (a) pictures, videos, or movies that depict actual naked individuals or persons having sexual intercourse; or (b) written or audio material that depicts naked individuals or persons having sexual intercourse.” Hentai pornography was defined as follows: “To use hentai means to intentionally view, read, or listen to (a) pictures, videos, or movies that depict nude animated characters, or animated characters having sex; or (b) written or audio material that depicts nude animated characters, or animated characters having sex.” These definitions were adapted from the International Sex Survey protocol (Bőthe et al., 2021).

The frequency of pornography use for both types was measured through the question “In the last year (last 12 months), how often did you use hentai/human pornography?,” with 11 possible answers ranging from “*never*” to “*more than 7 times a week*.” This measure was turned into a dummy variable, to be used in mediating/regression models. For hentai pornography, the cut point was set at the 50th percentile (0 = “*Once in the last year*” to “*Once a month*”; 1 = “*2–3 times a month*” to “*More than* *7 times in a week*”). For human pornography, the cut point was also set at the 50th percentile (0 = “*Once in the last year*” to “*2–3 times a month*” ; 1 = “*Once a week*” to “*More than 7 times in a week*”).

Furthermore, the intensity of pornography use was assessed via “When you use hentai/human pornography, how long do you spend with it per session?” with an open answer format in minutes. Similarly, this measure was also turned into a dummy variable with the cut point at the 50th percentile for hentai pornography (0 = *equal or less than 15* *min*; 1 = *over 16* *min*) and for human pornography (0 = *equal or less than 15* *min*; 1 = *over 16* *min*).

#### Sexually Aggressive Behaviors Scale

The SABS is a self-report scale with 26 items, 10 of them being critical items and 16 being filling items (used to conceal the relevant items), that assess the lifelong frequency of attempted aggressive sexual interaction (i.e., sexual coercion, sexual abuse, and physical force; Anderson, 1998). In each item, participants are questioned about how many times they have initiated sexual contact in each situation, for example, “How many times have you attempted to have sexual contact with a woman/man by pressuring her/him with verbal arguments?,” “How many times have you attempted to have sexual contact with a woman/man by threatening to use some degree of physical force (holding her/him down, hitting her/him, etc.)?.” The items were dichotomized, with zero meaning never having conducted such behavior, and one indicating that the behavior has occurred at least once.

This scale has been validated for the Portuguese population, with both male and female college student samples (Moreira et al., 2022); the Portuguese version is one-dimensional, consisting of a latent factor with 10 items named “Sexual Aggression” (Rosa et al., 2024). The present work revealed a KR-20 coefficient of .86 for the male SABS. For the female SABS, the KR-20 score was .67.

#### Scale of Beliefs About Sexual Violence

The Scale of Beliefs About Sexual Violence (ECVS; Martins et al., 2012) is a self-report scale, developed for the Portuguese population, composed of 30 items that measure the degree of acceptance of prejudicial beliefs about sexual violence, such as rape myths, for example, “Forcing your spouse to have sexual relations isn’t rape,” “Some people secretly want to be raped and would like for it to happen.” The items are on a 5-point Likert scale, ranging from 1 = *completely disagree* to 5 = *completely agree*. The original scale presents good psychometrics properties (Martins et al., 2012); in the current work, the Cronbach alpha was excellent (α = .94), according to the criteria by George and Mallery (2010).

### Procedure

To test our hypotheses, a set of mediation analyses was conducted utilizing the frequency and intensity with which hentai pornography is used as a predictor of sexually aggressive strategies; the endorsement of rape myths was established as the mediator. For this purpose, model 4 in the PROCESS macro tool for SPSS by Hayes (2013) was used. Four models were tested. The first two have the frequency of hentai pornography use as the predictor variable (one mediating model per gender). The other two models have the intensity of hentai pornography use as the predictor variable (one mediating model per gender).

The analyses were conducted on IBM SPSS Statistics 27. Statistical significance was assumed if zero was not contained within the confidence intervals, or if the *p* value was under .05 (Prel et al., 2009).

## Results

### Hentai Pornography

To start the study, bivariate correlations were conducted to establish associations between predictors, mediators, and outcome variables. In male participants, correlations were identified between all variables with the exception of intensity of hentai use and sexually aggressive behaviors. In female participants, the intensity of hentai use doesn’t show a correlation with sexually aggressive behaviors (SABS) and rape myths (ECVS), but correlations exist between the other variables. As stated by Agler and De Boeck (2017), the mediator can mediate the association between the predictor and the dependent variable even if the total effect is not significant. PROCESS (Hayes, 2013) uses a bootstrapping method to determine the confidence intervals for the indirect effect, thus being more rigorous than the causal steps approach. See Table 2 for the correlation results in male and female participants.

**Table 2.** Pearson Coefficient for Correlations Between Variables of Interest in Hentai Pornography.

Gender	Frequency	Intensity	SABS	ECVS
Male				
Frequency	-	.208**	.126**	.149**
Intensity	.208**	-	.012	.089*
SABS	.126**	.012	-	.476**
ECVS	.149**	.089*	.476**	-
Female				
Frequency	-	.295**	.145*	.153*
Intensity	.295**	-	.028	.062
SABS	.145**	.028	-	.568**
ECVS	.153*	.062	.568**	-

Note. ECVS = Scale of Beliefs About Sexual Violence; SABS = Sexually Aggressive Behaviors Scale.

* Correlation is significant at the .05 level (two-tailed).

** Correlation is significant at the .01 level (two-tailed).

#### Men

A significant relationship between the frequency of hentai pornography use and sexually aggressive behavior was found (*B* = 0.40, *SE* = 0.15, 95% CI [0.111, 0.699]). After controlling for the mediator, rape myths endorsement, the direct effect of the frequency of hentai use on sexually aggressive behavior was no longer significant (*B* = 0.17, *SE* = 0.13, 95% CI [−0.089, 0.428]). The mediation analysis was statistically significant (*B* = 0.24, *SE* = 0.10, 95% CI [0.072, 0.452]), and accounts for a full mediation. The percentage of explained variance of the model was 25% (*R*^2^ = .25).

Regarding the intensity of hentai pornography use, it was not a statistically significant predictor of sexually aggressive strategies (*B* = 0.04, *SE* = 0.13, 95% CI [−0.226, 0.298]). However, the intensity of hentai use was a predictor of rape myth endorsement (*B* = 3.51, *SE* = 1.73, 95% CI [0.114, 6.908]), which in turn successfully predicted sexually aggressive strategies (*B* = 0.04, *SE* = 0.003, 95% CI [0.031, 0.043]). An indirect effect was found between hentai pornography use and sexually aggressive strategies when inserting rape myths endorsement as a mediator (*B* = 0.13, *SE* = 0.08, 95% CI [0.004, 0.295]). The percentage of explained variance of the model was 23% (*R*^2^ = .23).

#### Women

The frequency of hentai pornography use significantly predicted sexually aggressive behavior (*B* = 0.45; SE = 0.18, 95% CI [0.086, 0.809]). After controlling for rape myths endorsement, the direct effect of the frequency of hentai use on sexually aggressive behavior was no longer significant (*B* = 0.17, *SE* = 0.15, 95% CI [−0.127, 0.463]). The indirect effect, using rape myths endorsement as a mediator, was statistically significant (*B* = 0.28, *SE* = 0.18, 95% CI [0.004, 0.714]), and signified a full mediation. The percentage of explained variance of the model was 37% (*R*^2^ = .37).

The intensity of hentai pornography use was not a statistically significant predictor of sexually aggressive strategies (*B* = 0.07, *SE* = 0.14, 95% CI [−0.200, 0.347]) nor rape myth endorsement (*B* = 1.47, *SE* = 1.26, 95% CI [−1.011, 3.955]), which precludes a mediation relationship.

See Table 3 for the non-standardized regression coefficients, and lower and upper limits of the 95% CI, for the direct, indirect, and total effects, in male and female participants.

**Table 3.** Non-Standardized Regression Coefficients, and Lower and Upper Limits of the 95% CI for Male and Female Participants That Use Hentai Pornography.

Gender	*B*	*SE B*	95% CI
Male			
Frequency			
a	6.08	1.90	[2.349, 9.803]
b	0.04	0.003	[0.032, 0.045]
c′	0.17	0.15	[−0.127, 0.463]
c	0.40	0.15	[0.111, 0.699]
ab	0.24	0.05	[0.072, 0.452]
Intensity			
a	3.51	1.73	[0.114, 6.908]
b	0.04	0.003	[0.032, 0.045]
c′	−0.09	0.12	[−0.325, 0.137]
c	0.04	0.13	[−0.226, 0.298]
ab	0.13	0.08	[0.005, 0.295]
Female			
Frequency			
a	4.26	1.66	[0.992, 7.524]
b	0.07	0.01	[0.055, 0.076]
c′	0.17	0.13	[−0.089, 0.428]
c	0.45	0.18	[0.086, 0.809]
ab	0.28	0.18	[0.004, 0.714]
Intensity			
a	1.47	1.26	[−1.011, 3.955]
b	0.06	0.005	[0.055, 0.074]
c′	−0.02	0.11	[−0.244, 0.202]
c	0.07	0.14	[−0.199, 0.347]
ab	0.07	0.06	[−0.057, 0.292]

*Note.* a = direct effect hentai pornography use—rape myths endorsement; b = direct effect rape myths endorsement—sexually aggressive behavior; c′ = hentai pornography—use sexually aggressive behavior; c = total effect; ab = indirect effect/mediation model.

### Human Pornography

Bivariate correlations were conducted to establish associations between the frequency and intensity of human pornography use, belief in rape myths, and sexually aggressive behaviors. See Table 4 for the correlation results in male and female participants.

**Table 4.** Pearson Coefficient for Correlations Between Variables of Interest in Human Pornography.

Gender		Frequency	Intensity	SABS	ECVS
Male	Frequency	-	−.026	.047	.074
	Intensity	−.026	-	.015	.013
	SABS	.047	.015	-	.481**
	ECVS	.074	.013	.481**	-
Female	Frequency	-	.115**	.109**	.149**
	Intensity	.115**	-	.040	.011
	SABS	.109**	.040	-	.487**
	ECVS	.149*	.011	.487**	-

Note. ECVS = Scale of Beliefs About Sexual Violence; SABS = Sexually Aggressive Behaviors Scale.

* Correlation is significant at the .05 level (two-tailed). **Correlation is significant at the .01 level (two-tailed).

#### Men

No significant relationship was found between the frequency of human pornography use and sexually aggressive behavior (*B* = 0.15, *SE* = 0.12, 95% CI [−0.090, 0.399]), nor between the frequency of human pornography use and endorsement of rape myths (*B* = 3.14, *SE* = 1.62, 95% CI [−0.045, 6.327]), thus mediation was not possible.

Regarding the intensity of human pornography use, it didn’t predict the report of sexually aggressive strategies (*B* = 0.04, *SE* = 0.11, 95% CI [−0.171, 0.259]). Additionally, there wasn’t an indirect effect between human pornography use and sexually aggressive strategies when inserting rape myths endorsement as a mediator (*B* = 0.02, *SE* = 0.05, 95% CI [−0.080, 0.135]).

#### Women

The frequency of human pornography use significantly predicted sexually aggressive behavior (*B* = 0.29; *SE* = 0.11, 95% CI [0.078, 0.499]). After controlling for rape myths endorsement, the direct effect of the frequency of human pornography use on sexually aggressive behavior was no longer significant (*B* = 0.09, *SE* = 0.09, 95% CI [−0.091, 0.280]). The indirect effect, using rape myths endorsement as a mediator, was statistically significant (*B* = 0.19, *SE* = 0.11, 95% CI [0.034, 0.445]), and signified full mediation. The percentage of explained variance of the model was 1.2% (*R*^2^ = .012).

The intensity of human pornography use was not a statistically significant predictor of sexually aggressive strategies (*B* = 0.09, *SE* = 0.09, 95% CI [−0.082, 0.256]). Additionally, the indirect effect was not statistically significant (B = 0.12, *SE* = 0.04, 95% CI [−0.064, 0.112]).

See Table 5 for the non-standardized regression coefficients, and lower and upper limits of the 95% CI, for the direct, indirect, and total effects, in male and female participants.

**Table 5.** Non-Standardized Regression Coefficients, and Lower and Upper Limits of the 95% CI for Male and Female Participants That Use Human Pornography.

Gender	*B*	*SE B*	95% CI
Male			
Frequency			
a	3.14	1.62	[−0.045, 6.327]
b	0.04	0.003	[0.032, 0.042]
c′	0.04	0.11	[−0.176, 0.252]
c	0.15	0.12	[−0.090, 0.399]
ab	0.12	0.06	[0.007, 0.258]
Intensity			
a	0.51	1.44	[−2.314, 3.328]
b	0.04	0.003	[0.032, 0.042]
c′	0.03	0.10	[−0.163, 0.213]
c	0.04	0.11	[−0.171, 0.259]
ab	0.01	0.04	[−0.080, 0.135]
Female			
Frequency			
a	3.88	1.05	[1.813, 5.938]
b	0.05	0.004	[0.043, 0.057]
c′	0.09	0.09	[−0.091, 0.280]
c	0.29	0.11	[0.078, 0.499]
ab	0.19	0.11	[0.033, 0.445]
Intensity			
a	0.23	0.85	[−1.433, 1.898]
b	0.05	0.004	[0.043, 0.057]
c′	0.08	0.07	[−0.072, 0.222]
c	0.09	0.09	[−0.082, 0.256]
ab	0.01	0.04	[−0.064, 0.112]

Note. a = direct effect human pornography use—rape myths endorsement; b = direct effect rape myths endorsement—sexually aggressive behavior; c′ = human pornography—use sexually aggressive behavior; c = total effect; ab = indirect effect/mediation model.

## Discussion

Pornography consumption is argued to increase the likelihood of endorsing sexually aggressive attitudes (Hald et al., 2010; Wright et al., 2016), making individuals more susceptible to perpetrating various forms of sexual aggression. These behaviors include verbal or physical coercion. Exposure to a specific type of pornography, hentai, is argued to contain themes and elements that affect an individual’s susceptibility toward engaging in acts of sexual aggression (Ferguson & Hartley, 2022; Harrington & Neilson, 2009).

Against this background, the present work aimed to provide insight into the relationship between hentai pornography use, with the frequency and intensity of hentai pornography use as predictors, and sexually aggressive strategies, mediated by the endorsement of rape myths, in male and female college students. The findings necessitate careful consideration owing to the constraints inherent in the present investigation. Presently, we contemplate the research methodology, highlighting both its limits and ramifications on result interpretation. In closing, several recommendations are presented for future investigation.

Considering our hypotheses, while the relationship between the frequency of use of hentai pornography and sexually aggressive behavior was found in both genders, the intensity of hentai use had no direct link to sexually aggressive behaviors.

Findings revealed that the frequency of hentai pornography use predicts rape myths endorsement. Literature had previously found a positive correlation between the use of pornography and rape myths acceptance (Foubert et al., 2011) as well as attitudes supporting violence against women (Hald et al., 2010; Malamuth et al., 2012). These findings can be extended to hentai as it is a type of pornography. While there is little research concerning the effects of the different factors of pornography use, literature leads to the expectation that higher frequency is related to more sexually aggressive behaviors (de Heer et al., 2021) A potential explanation for this could be possible desensitization resulting from overexposure to pornography (Linz, 1985).

The intensity in which someone uses hentai does not predict sexually aggressive behaviors in any gender. However, the intensity of hentai use has an effect on sexually aggressive behavior through the endorsement of rape myths.

Rape myths acceptance is beneficial to sexually aggressive individuals (Burgess, 2007), as people who adhere to rape myths minimize sexual violence and discredit victims. Unsurprisingly, rape myths endorsement has been associated with sexual aggression perpetration (Forbes et al., 2004). These types of beliefs often regard women as the victims and are associated with attitudes supporting violence against women (Hald et al., 2010; Malamuth et al., 2012). However, the present research found an association between rape myths endorsement and sexually aggressive behavior in both male and female participants.

As hypothesized, the expected link between hentai pornography use and sexually aggressive tendencies was better explained by the endorsement of rape myths. Full mediations were found between the frequency of hentai use and sexually aggressive behavior in both genders. Despite the absence of a total effect between the intensity of hentai use and sexually aggressive behavior in male participants, the intensity of use predicted the belief in rape myths, which in turn predicted sexually aggressive behaviors. Furthermore, there was a significant mediation relationship found. There is not enough evidence to how the intensity of hentai use influences sexually aggressive behaviors, but if this influence exists, it is likely due to the intervening effect of rape myths endorsement.

This same phenomenon does not occur in female participants, resulting in gender differences concerning the intensity criteria. Possible explanations could be that female participants exhibit more consistent levels of intensity, or that those levels are lower and thus irrelevant to the other variables.

These results build on existing evidence from studies by Malamuth (2018) and Vega and Malamuth (2007), in which the connection between pornography use and sexual violence was found in a male sample but only in individuals at higher risk of committing sexual violence. This risk was assessed through their inclination toward hostile masculinity characteristics (which included belief in rape myths) and attitudes toward impersonal sex. The Confluence model by Malamuth et al. (2021) also concludes that factors such as “extreme pornography use” are secondary to the other key risk factors, such as “Impersonal Sex” and “Hostile Masculinity,” thus only influencing the individual’s actions if the core factors are already present. The use of human/realistic pornography was also analyzed to add to a more in-depth discussion. Our findings revealed that the frequency of human pornography use is associated with sexually aggressive behaviors, exclusively in female college students. In contrast, when examining the effects of hentai pornography use, we observed that the frequency of use is associated with sexually aggressive behaviors in both genders. In agreement with hentai pornography, the intensity of use is not associated with sexually aggressive behaviors in any gender. Moreover, we investigated whether the belief in rape myths as a mediator could better explain these relationships. We found that it only mediates the relationship between human pornography use and sexually aggressive behaviors in female college students. There is an indirect effect between those same variables in male college students, but it is not enough for a mediation, as the frequency of use does not predict rape myths endorsement.

As such, the biggest difference between hentai and human pornography is in the frequency with which male college students use them, as intensity does not show mediation in any case. These outcomes are partially in line with a study by Park et al. (2022) that compared hentai pornography users with human pornography and didn’t find many significant differences. Still, in our study, hentai pornography-rape myths’ interaction role in sexual aggression seemed to have had a greater role, as seen by higher explained variance in the mediation models as compared with the models pertaining to human pornography.

The findings obtained in this work contribute to the lesser explored genre of hentai pornography, by showing a novel perspective on its connection with sexually aggressive behavior in its target audience, age-wise.

This work also reinforces conclusions in previous studies establishing links between pornography use and rape myth beliefs (Burgess, 2007; Foubert et al., 2011), while identifying new associations related to the genre of hentai. While clarifying these associations, this work reveals that mediations play a crucial role in connecting hentai use, rape myths, and sexual violence in college students, which opens doors for future investigation into other mediators involved in this relationship and its possible replicability in broader populations.

However, no study is without its limitations. First, it is impossible to infer that the influence of hentai pornography in the mediation analyses is solely due to the hentai genre rather than the use of pornography as a whole. Furthermore, the data has a correlational nature, so causal inferences cannot be made. Additionally, when tackling sexual aggression survey participants are susceptible to the influence of social desirability. It is expected that themes relating to sexuality and intimacy raise sensitivity issues in respondents, which could explain the high rate of dropouts in this study. Concomitantly, while it is likely that people underreported sexually aggressive attitudes they might have had, it seems possible that people also overemphasized them, as well as embellished the frequency and intensity in which they used pornography, due to college students often participating in Internet mischief. Future research can minimize this effect by utilizing a social desirability measure and implementing attention checks. The sampling method can also create self-selection bias, as voluntary response samples generally attract people who are more likely to answer surveys and have stronger feelings about the theme being investigated, thus not being fully representative of the population. All measures utilized in this study were of the self-report kind, making the data vulnerable to reporting bias (Gomes et al., 2019).

Another inherent constraint of this study is the absence of comprehensive data regarding hentai pornography, as well as its possible influence on those who view it, which hinders researchers from completely understanding how it influences sexually aggressive attitudes. However, this work aims to contribute to this gap in the literature and to the limited research concerning the intensity with which any pornography is viewed and how it impacts the individual, as well as building on studies that study the differences between hentai and human pornography users.

Additionally, the generalizability of the results is limited by the narrow scope focused on college students who identified as heterosexuals, so generalizing the findings to sexual and gender minorities or the population outside of higher education is not possible. The heterosexual population was studied due to the SABS only having been validated for that population. To address these constraints, future research ought to consider the limitations of using measures that were initially designed for application in heterosexual populations when examining sexual aggression and victimization across heterogeneous populations, in this case, the SABS. This could entail using alternative scales or validating existing ones with sexual and gender minorities to encompass all individuals. By resolving this restriction, future studies can attain a better comprehension of the sexually aggressive attitudes of people toward the same sex.

Future studies should also aim to include using different measures of hentai pornography use or even different frequency and intensity measures or scales, to check for result variability depending on the choice of measures. Further research on hentai pornography is necessary to address the current scarcity of knowledge and gain a deeper understanding of this consistently popular genre of pornography.

This work established relationships that can be addressed and prevented early on in a person’s life to prevent possibly aggressive sexual behaviors, as well as other adverse effects of ignorance about sexual violence.

## Conclusion

This research aimed to identify how hentai pornography consumption influenced sexually aggressive strategies. Based on a quantitative analysis of sexuality in college students, it can be concluded that the frequency in which one uses hentai pornography predicts sexually aggressive strategies, and that the endorsement of rape myths is an important factor in the relationship between the frequency of hentai pornography use and the use of sexually aggressive strategies, and between the intensity of hentai pornography use and sexually aggressive strategies.

To conclude, this work paves the way for additional research into the impact of animated and fantastical pornography in a population where sexual violence is rampant. It provides researchers with insight into this genre of pornography, as well as what aspects of its use could be relevant in predicting sexual aggression. This study further supports existing evidence connecting pornography use with sexually aggressive behaviors and rape myths endorsement, strengthening the growing body of literature in favor of the existence of these relationships.
